# Idiopathic Unilateral Adrenal Haemorrhage and Adrenal Mass: A Case Report and Review of the Literature

**DOI:** 10.1155/2013/567186

**Published:** 2013-04-10

**Authors:** Christos Christoforides, Athanasios Petrou, Marios Loizou

**Affiliations:** Department of Surgery, Nicosia General Hospital, 29 Patmos Street, Strovolos, 2062 Nicosia, Cyprus

## Abstract

We report an unusual case of idiopathic unilateral adrenal haemorrhage (AH) in a 55-year-old patient. This rare case had two characteristics that made it worth of report. First, idiopathic adrenal haemorrhage is very uncommon, and second it was presented as a huge, 23 cm diameter and 2,123 gr weight, “silent” adrenal mass. It is important to distinguish a benign lesion like this from a neoplasm, although we were not able to identify it preoperatively and the diagnosis was only made after the excised specimen was examined by a group of experienced histopathologists. Only a few similar published cases, to our knowledge, are described in the worldwide literature and even fewer of this size.

## 1. Introduction

Adrenal haemorrhage (AH) is an uncommon condition more frequently presented in adolescents than in adults (7 : 1). Symptoms and physical findings are not specific and vary among patients. Idiopathic, unilateral AH is a rare entity that either may have an acute presentation (e.g., idiopathic adrenal rupture) or may present as an asymptomatic adrenal mass, as has been reported by other series [1, 2]. Most authors concluded that it is usually caused by blunt abdominal trauma (traumatic adrenal rupture), but it also has occurred in liver transplant recipients and in patients with primary adrenal or metastatic tumors. Infrequently, unilateral adrenal haemorrhage is associated with otherwise uncomplicated pregnancy, neurofibromatosis 1, or long-term nonsteroidal anti-inflammatory drug (NSAID) use [3–6]. In the present study, we report the case of a 55-year-old patient with idiopathic unilateral adrenal haemorrhage, presenting as a huge adrenal mass.

## 2. Case Report

The patient was admitted to our department for the evaluation of a palpable mass in the right side of his abdomen. His past medical history and any medication he ever had were irrelevant with the current problem. Physical examination revealed a palpable mass, approximately 20 × 20 cm in size, occupying an area from the upper to the lower right abdominal quadrantus. No pain or tenderness around the mass was reported and the patient mentioned only some discomfort when he was lying at night. 

His vital signs were within the normal range and the laboratory data showed mild anaemia (haemoglobin: 10.9 g/dL), WBC 8190 × 10^9^/L, without any hepatic, renal, or adrenal dysfunction from the blood or urine examinations he had. 

Moreover, his serum tumor markers and coagulation function were all within normal values. 

Contrast-enhanced computed tomography (CT) showed a huge, well-circumscribed, 23 × 20 cm, cystic lesion between liver and right kidney with the enhancement of the capsule around the tumor. 

**Figure 1:**
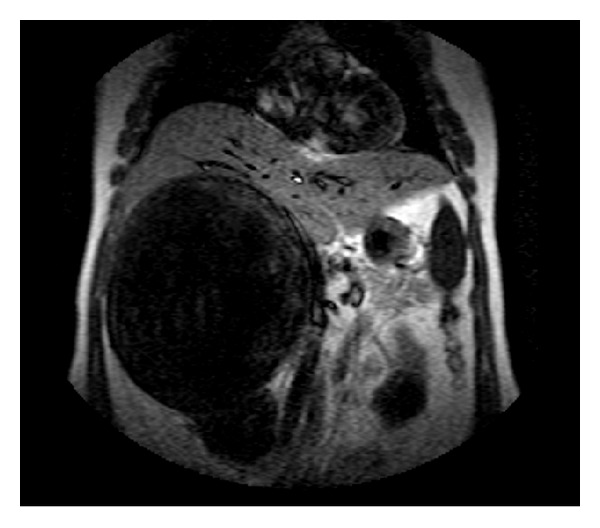
Coronal plane of patients preoperative MRI scan, demonstrating the right quadrant huge mass.

**Figure 2:**
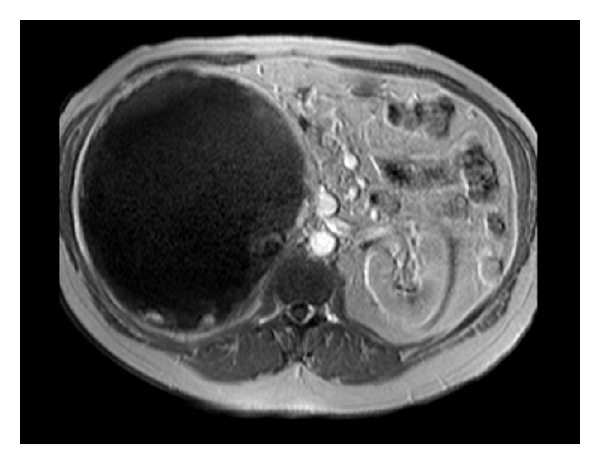
Patient's MRI T1-weighted image with characteristic high-intensity signal from the mass.

**Figure 3:**
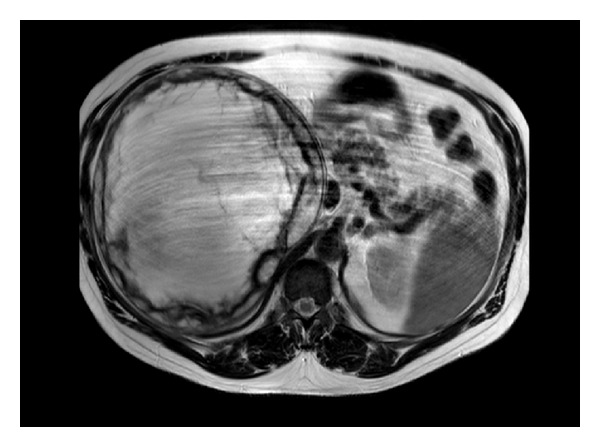
Patient's MRI T2-weighted image with heterogeneously low signals.

 No liver metastasis or lymph node swelling was noted. The gadolinium-enhanced magnetic resonance imaging (MRI) that followed showed a huge multicystic lesion, in the region of the right adrenal gland, with a thick capsule containing several septations especially in the periphery of the lesion (Figures 1, 2, and 3). 

 The abnormality deviated the liver upwards, the right kidney downwards and posteriorly, the pancreas to the left, and the abdominal aorta and IVC were deviated to the left. The vascularity of the lesion was not clearly demonstrated, but there were large venous structures peripheral to the lesion draining into the IVC. 

The previously described findings were discussed in the MDT meeting and the decision for surgery was made.

**Figure 4:**
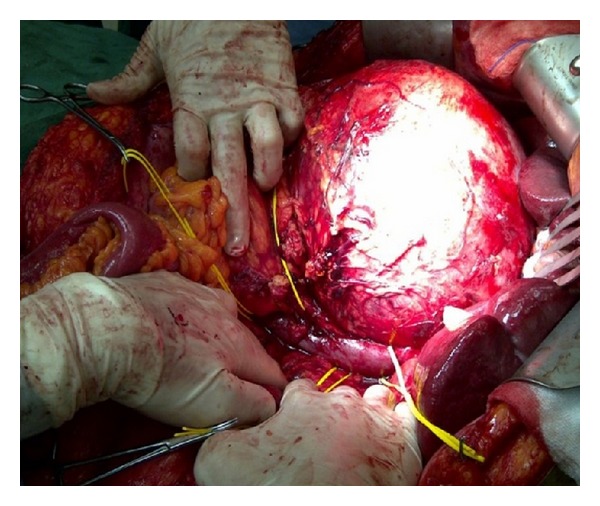
Intraoperative image showing the close relation between IVC and the mass.

**Figure 5:**
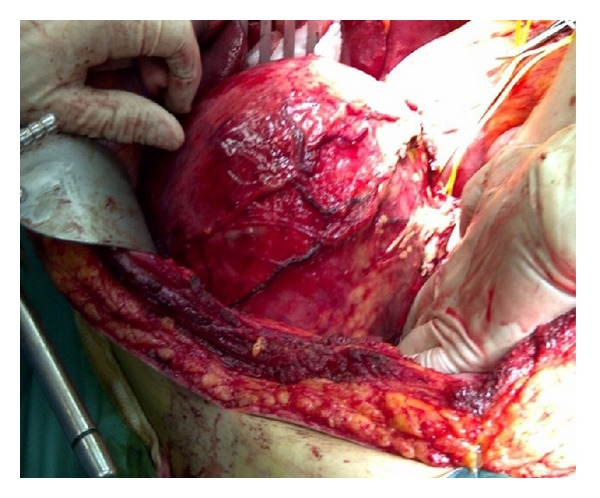
Intraoperative image during lesion dissection.

During the 3-hour exploratory laparotomy that followed the lesion was carefully dissected from the surrounding structures (i.e., liver, pancreas, and right kidney) and excised along with the surrounding fatty tissues. The lesion was firmly attached to the IVC and right renal vein, so a full isolation of both was necessary and successfully performed (Figures 4 and 5). Postoperatively, the patient was transferred to the surgical ward in a stable condition. The postoperative period was uneventful and the patient was discharged in a good condition on the 6th postoperative day. 

The histopathology report described a yellow-whitish lesion with traces of normal adrenal gland tissue and appearances of adrenal haemorrhage. They reported that the lesion was consisted entirely of blood clots, fibrinoid debris, and ghost outlines of proexisting vessels. Finally, they noted that it was covered by a fibrous pseudocapsule and no evidence of malignancy was noted. The final diagnosis for this patient was idiopathic adrenal haemorrhage.

## 3. Discussion

Adrenal haemorrhage is a rare yet potentially life-threatening event that occurs both in traumatic conditions and in a variety of nontraumatic conditions. To our knowledge, it is an autopsy finding in most of the reported cases [3]. It may be either unilateral or bilateral. In one of the biggest published series, Vella et al. have summarized 141 cases of AH in a 25 years experience period at the Mayo Clinic [7]. They classified AH into the following seven categories: incidentaloma (28 cases), spontaneous AH (16 cases), AH associated with antiphospholipid- and heparin-associated thrombocytopenia (20 cases), postoperative AH (14 cases), AH associated with anticoagulation therapy (3 cases), AH associated with trauma (4 cases), and AH associated with sepsis (especially meningococcemia) or severe stress (56 cases). Our present case is an example of idiopathic/spontaneous AH. The incidence of idiopathic adrenal haemorrhage in some published autopsy series is very low and reaches 1.1% in some of the biggest (>25000 cases) autopsy series [3, 7–11].

Clinical manifestations of adrenal haemorrhage can vary widely depending on the degree and rate of haemorrhage, as well as the amount of adrenal cortex compromised by haemorrhage, as similar cases have reported [11–13]. Although an isolated focal unilateral adrenal haemorrhage may present subclinically, there are reports of massive bilateral adrenal haemorrhage which led to rapid cardiovascular collapse and ultimate death because of being misdiagnosed and not treated quickly [14].

According to the underlying cause, clinical presentation varies and symptoms may include abdominal or flank pain, nausea, vomiting, weakness, weight loss, or mental confusion (adrenal insufficiency). Those are often accompanied with physical findings of fever, hypotension, abdominal tenderness or distension, and a palpable abdominal mass, as discussed elsewhere [2, 11].

The laboratory findings also vary and may include a falling haematocrit level, leukocytosis, or electrolytes abnormalities [15, 16]. Some authors concluded that diagnosis of adrenal haemorrhage is often complicated by its nonspecific presentation and tendency to occur in the setting of acute illness and other complicating medical conditions [17]. In other reported cases of a huge adrenal mass as in our patient, the differential diagnosis includes a number of varieties such as adrenal incidentaloma, pheochromocytoma, adrenocortical carcinoma, angiomyolipoma, and collision tumor [13]. The first thing to determine is whether the mass is functioning or not functioning. Functional tumors may cause Cushing's disease from hypercortisolemia, hyperaldosteronemia, and Conn's syndrome, or they may be virilizing tumors from steroid overproduction. Pheochromocytoma is also a primary concern when evaluating functional adrenal masses because of the potential hazards of massive catecholamine release. Nonfunctioning tumors vary significantly in appearance and presentation, and, due to that, the preoperative diagnosis is often missed, as others support [11, 18].

Imaging assists and complements the clinical and endocrine evaluation of adrenal masses. Various studies showed that, although ultrasonography is a fast, low-cost, and widely available method, it is usually limited to infants and children [19, 20]. Computed tomography (with or without IV contrast) is the cornerstone of imaging studies. It provides information regarding the homogeneity, size, presence of calcifications, extend of local invasion (in case of neoplasms), and areas of necrosis. CT can demonstrate adrenal masses >5 mm in diameter, but the most widely accepted threshold is 4 cm, with 90% sensitivity but low specificity. The majority of the existing literature supports that changes in size, shape, and radiograph absorption on unenhanced CT are useful information in predicting potential malignancy in such a mass [12, 19, 21, 22]. Others suggested magnetic resonance imaging (MRI) as a more accurate and safe (does not expose patient to ionizing radiation) imaging modality for diagnosing adrenal haemorrhage or haematoma, with high signal intensity on T1-weighted images. MRI also may differentiate subacute from chronic haemorrhage, but still remains an expensive and not always available imaging modality [3, 21, 22]. Some authors add adrenal scintigraphy to the imaging studies available during the investigation of such masses. Unlike other imaging, it can determine if there is extra-adrenal disease or whether a tumor is functioning. Sensitivity ranges from 77% to 89% and specificity from 88% to 100%, but radiation exposure, high cost, and limited availability restrict its use [22].

 Some have proposed a descent approach to incidentally discovered adrenal mass, which includes assessing the mass size, determination of whether is solid or cystic, and excluding biochemically active tumors by all the appropriate laboratory studies [8, 23]. There are also recommendations (the NIH state-of-the-science conference statement) supporting that because benign adrenal neoplasms bigger than 6 cm are rare, solid, or cystic without clear view, adrenal masses larger than 6 cm should be surgically excised after studies of biochemical activity [10, 23]. According to these recommendations, our patient had an exploratory laparotomy with the unexpected finding that we report. From our review of the literature, we found only a few similar cases of idiopathic adrenal haemorrhage that had been diagnosed before death and treated surgically. The majority of those patients had significant abdominal pain, and the diagnosis was made at the time of the operation [1, 5, 6, 24–26]. None of them is present in our case. The operative findings and the preoperative “silent” clinical presentation of such a rare entity should make us always have in mind that even the least possible diagnosis can be found in such patients [11].

## 4. Conclusion

Adrenal haemorrhage is a rare cause of adrenal mass and is generally associated with trauma, infection, or bleeding diathesis. Only a few isolated cases of similar pathology (idiopathic adrenal haemorrhage) exist in the literature and nearly all of them were postoperatively diagnosed as such. Though rare, signs of adrenal haemorrhage may include an asymptomatic adrenal mass and should be considered in the differential diagnosis of this problem.
